# Supplementary material for: Identification of photoperiod-induced specific miRNAs in the adrenal glands of Sunite sheep (Ovis aries)
Source: Front Vet Sci. 2022 Jul 22;9:888207. doi: 10.3389/fvets.2022.888207 (PMC9354845; doi:10.3389/fvets.2022.888207)
Supplement: Supplementary file 1 [file Data_Sheet_1.ZIP › Supplementary file/miRDeep2 file.zip!/miRDeep2 file/Know_Structure/SPLP42A1/oar-mir-106b.pdf]

miRBase precursor	: oar-mir-106b
Total read count	: 3616
oar-miR-106b read count	: 1295
remaining reads	: 2321

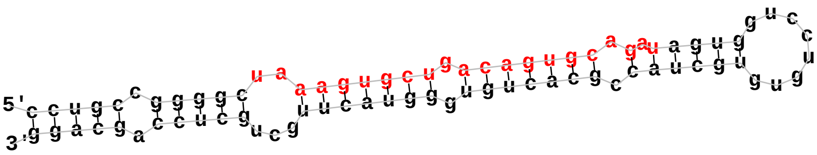

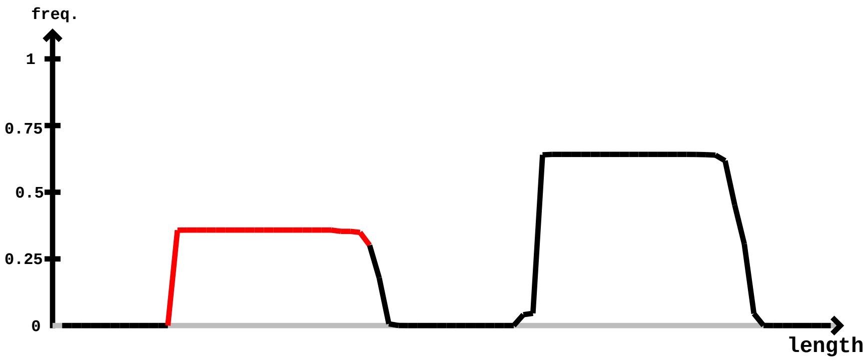


```
               oar-miR-106b
5'- ccugccggggcuaaagugcugacagugcagauaguggucccugugugcuaccgcacuguggguacuugcugcuccagcagg   -3'    exp
    (((((.(((((..(((((((.(((((((.(.(((((........)))))).))))))).)))))))...))))).)))))    reads      mm         sample
    ...........uaaagugcugacagugc....................................................    14         0          seq
    ...........uaaagugcugacagugA....................................................    3          1          seq
    ...........uaaagugcugacaguUc....................................................    1          1          seq
    ...........uaaagugcugacagugcag..................................................    14         0          seq
    ...........uaaagugcugacagugcaga.................................................    173        0          seq
    ...........uaaagugcugacagugcagau................................................    442        0          seq
    ...........uaaagugcugacagugcagaua...............................................    623        0          seq
    ...........uaaagugcugacagugcagauag..............................................    22         0          seq
    ...........uaaagugcugacagugcagauagu.............................................    1          0          seq
    ............aaagugcugacagugcaga.................................................    1          0          seq
    ............aaagugcugacagugcagau................................................    1          0          seq
    ...............................................uaccgcacuguggguacu...............    1          0          seq
    ...............................................uaccgcacuguggguacuu..............    1          0          seq
    ...............................................uaccgcacuguggguacuugc............    10         0          seq
    ...............................................uaccgcacuguggguacuugcu...........    134        0          seq
    ...............................................uaccgcacuguggguacuugcugc.........    1          0          seq
    ................................................accgcacuguggguacuugc............    2          0          seq
    ................................................accgcacuguggguacuugcu...........    7          0          seq
    ................................................accgcacuguggguacuugcug..........    6          0          seq
    ................................................accgcacuguggguacuugcugc.........    4          0          seq
    .................................................ccgcacuguggguacuu..............    2          0          seq
    .................................................ccgcacuguggguacuug.............    6          0          seq
    .................................................ccgcacuguggguacuugc............    64         0          seq
    .................................................ccgcacuguggguacuugcu...........    458        0          seq
    .................................................ccgcacuguggguacuugcug..........    522        0          seq
    .................................................ccgcacuguggguacuugcugc.........    935        0          seq
    .................................................ccgcacuguggguacuugcugcu........    161        0          seq
    .................................................ccgcacuguggguacuugcugcuc.......    1          0          seq
    ..................................................cgcacuguggguacuugcug..........    2          0          seq
    ..................................................cgcacuguggguacuugcugc.........    4          0          seq
```
